# Haplotype-resolved genome assembly of the tetraploid potato cultivar Désirée

**DOI:** 10.1038/s41597-025-05372-3

**Published:** 2025-06-20

**Authors:** Tim Godec, Sebastian Beier, Natalia Yaneth Rodriguez-Granados, Rashmi Sasidharan, Lamis Abdelhakim, Markus Teige, Björn Usadel, Kristina Gruden, Marko Petek

**Affiliations:** 1 National Institute of Biology, Department of Biotechnology and Systems Biology, Ljubljana, Slovenia; 2 Jožef Stefan International Postgraduate School, Ljubljana, Slovenia; 3 Institute of Bio- and Geosciences (IBG-4 Bioinformatics), Bioeconomy Science Center (BioSC), CEPLAS, Forschungszentrum Jülich GmbH, Jülich, Germany; 4 Plant Stress Resilience, Institute of Environmental Biology, Utrecht University, Utrecht, The Netherlands; 5 PSI (Photon Systems Instruments), Drásov, Czech Republic; 6 Molecular Systems Biology (MOSYS), Department of Functional and Evolutionary Ecology, University Vienna, Vienna, Austria; 7 Faculty of Mathematics and Natural Sciences, Institute for Biological Data Science, Cluster of Excellence on Plant Sciences (CEPLAS), Heinrich Heine University Düsseldorf, Düsseldorf, Germany

**Keywords:** Polyploidy in plants, Genome, Genomics

## Abstract

Cultivar Désirée is an important model for potato functional genomics studies to assist breeding strategies. Here, we present a haplotype-resolved genome assembly of Désirée, achieved by assembling PacBio HiFi reads and Hi-C scaffolding, resulting in a high-contiguity chromosome-level assembly. We implemented a comprehensive annotation pipeline incorporating gene models and functional annotations from the *Solanum tuberosum* Phureja DM reference genome alongside RNA-seq reads to provide high-quality gene and transcript annotations. Additionally, we provide a genome-wide DNA methylation profile using Oxford Nanopore reads, enabling insights into potato epigenetics. The assembled genome, annotations, methylation and expression data are visualised in a publicly accessible genome browser, providing a valuable resource for the potato research community.

## Background & Summary

Potato (*Solanum tuberosum*) is one of the most important and widely cultivated crops worldwide, with a significant role in global food security and agricultural research. Despite its significance, many studies still rely on the genome of the double monoploid (DM) clone of group Phureja DM1–3 516 R44^1,2^ which lacks a substantial portion of the gene repertoire and variability found in cultivated tetraploid potato varieties^3^.

The potato cultivar Désirée is a red-skinned late-season potato variety, originally bred in the Netherlands in 1962 by crossing parent cultivars Urgenta and Depesche (Potato Pedigree Database)^4^. It is still cultivated due to its favourable agronomic traits, such as predictable yields and high tolerance to drought and some pathogens^5^. It has also been used in breeding programs, yet a genome assembly for the Désirée cultivar has not been available. In research, it has been propagated in tissue cultures, and used for genetic manipulation including gene overexpression^6^, gene silencing^7^, and Crispr-Cas gene editing^8^.

Although haplotype-resolved genome assemblies are becoming common in diploid organisms, the high heterozygosity rate, extensive repeat content, and the autopolyploid nature of cultivated potatoes still present significant challenges for generating high-quality haplotype-resolved assemblies. Currently, five haplotype-resolved genomes of autotetraploid potato cultivars are publicly available^9–13^ as well as several phased diploid genomes^14–16^. The recently published haplotype-resolved tetraploid potato assemblies rely on labour-intensive techniques such as single-pollen sequencing^11^ or the use of parental and crossing material^12^, which may not always be available.

Adding to existing publicly available genomes, we provide a reference quality (CRAQ overall AQI of 97.5) haplotype-resolved genome assembly of the tetraploid cultivar Désirée, assembled using solely PacBio HiFi and Illumina Hi-C data. Our assembly is accompanied by a comprehensive structural and functional gene annotation reaching 99.4% BUSCO completeness for Solanaceae, accompanied by orthology to DM genes. For the potato research community, we provide an online resource featuring a genome browser (https://desiree.nib.si) and downloadable genomic assembly and annotation files, providing a valuable tool for studies involving allele-specific expression or promoter analysis.

## Methods

### Sample preparation and sequencing

Leaves from 4-week old *S. tuberosum* cv. Désirée plants were collected and flash-frozen. High molecular weight genomic DNA (HMW gDNA) used for PacBio HiFi, Illumina and Oxford Nanopore Technologies (ONT) sequencing was extracted from the leaf tissues using a modified CTAB method^17^. The concentration and quality of the extracted DNA were assessed using a NanoDrop spectrophotometer.

#### PacBio HiFi

HMW gDNA was sent to National Genomics Infrastructure (NGI) Sweden for library preparation and sequencing on the PacBio Sequel II platform. We obtained 79.4 Gbp of raw data, consisting of 4.1 million reads.

#### Illumina Hi-C

Leaves from 4-week old *S. tuberosum* cv. Désirée plants were collected, flash-frozen in liquid nitrogen and ground using mortar and pestle. Hi-C library prep using the Omni-C kit (Dovetail Genomics) and sequencing were performed on an Illumina NovaSeq 6000 platform by NGI Sweden. Sequencing generated 2018.4 million paired-end (2 × 150 bp) reads.

#### ONT

The HMW gDNA was used for ONT DNA library prep using the SQK-LSK110 kit and sequenced on a MinION using the FLO-MIN106 flow cell. Reads were basecalled using Dorado (v0.7.2) with the model dna_r9.4.1_e8_sup@v3.3 which generated 5.8 Gbp. The reads with methylation-related tags were converted to bedMethyl format using modkit (v0.4.1).

#### Illumina short reads

Illumina short-read library was constructed from the HMW gDNA and sequenced on Illumina NextSeq 2000 by ELIXIR Slovenia node to generate 150 bp paired-end reads. The short-read sequencing generated approximately 138 Gbp of raw data, consisting of 460.1 million paired-end (2 × 150 bp) reads.

### Genome size and heterozygosity estimation

The genome characteristics of *S. tuberosum* cv. Désirée, including genome size, heterozygosity, and repeat content, were estimated using Illumina short-read data and a k-mer based approach. A 21-mer frequency distribution was generated with Jellyfish (v2.2.10), and the genome’s key features were inferred using GenomeScope2 (v2.0). The haploid genome size was estimated at 669.6 Mbp, with a heterozygosity rate estimated at 3.8–5.7%.

### *De novo* genome assembly, Hi-C scaffolding and quality assessment

**Fig. 1:**
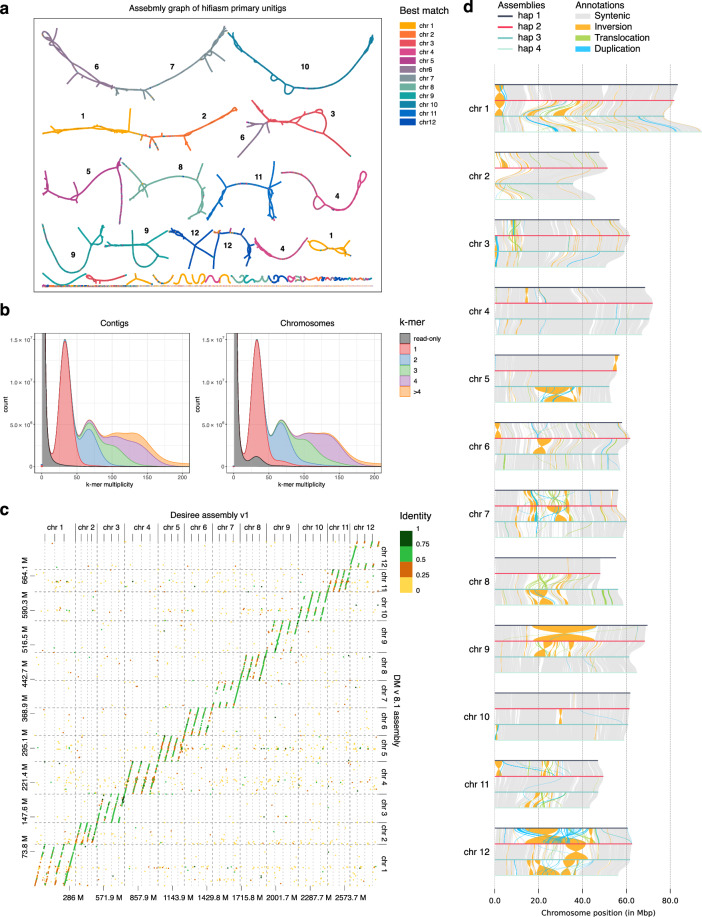
General characteristics of Désirée genome assembly (**a**) Assembly graph of primary unitigs coloured by best match to DM chromosomes (also designated with numbers on the graph). (**b**) Merqury k-mer spectra for initial contigs and scaffolded chromosomes. The k = 21 was used. K-mers are categorized as read-only (grey), unique (red), and shared (blue, green, purple, orange). Peaks corresponding to higher multiplicities indicate the presence of highly repeated k-mers. (**c**) Dot plot comparing cv. Désirée chromosome-anchored contigs with DM v8.1 chromosomes. The colour designates contig identity. (**d**) Genomic synteny of cv. Désirée haplotype-resolved assembly.

PacBio HiFi and Illumina Hi-C reads^18^ were initially assembled into four sets of haplotype-resolved contigs using Hifiasm (v0.19.8-r603)^19–21^. Hifiasm primary unitigs were searched against DM genome assembly with blastn (v2.5.0)^22^ and best matches were visualised on Graphical Fragment Assembly with Bandage (v0.8.1, Fig. 1a)^23^. We performed quality control of the contigs using Merqury (v1.3, Fig. 1b)^24^ k-mer spectra and BUSCO completeness scores (v5.4.7, solanales_odb10 dataset)^25^. The length of haplotype draft assemblies ranged from 761.6 Mbp to 888.4 Mbp with contig N50 sizes ranging from 7.0 Mbp to 13.7 Mbp (Table 1).

**Table 1:** Summary of the four haplotypes of the Désirée genome assembly.

	haplotype 1	haplotype 2	haplotype 3	haplotype 4	all haplotypes
Genome length (Mb)	888.4	862.7	761.6	858.5	3371.2
GC content (%)	35.31	35.27	35.12	35.47	35.3
Contig N50 (Mb)	11.5	13.7	11.7	7.0	10.8
Number of contigs	1126	867	1048	2695	5736
Chromosome length (Mb)	721.9	729.9	698.5	709.4	2859.6
Scaffold N50 (Mb)	56.9	61.4	60.1	57.1	58.0
Number of scaffolds	705	496	523	1350	3074
**Scaffolds BUSCO completeness (%)**					
Complete	96.2	96.1	96.6	95.7	99.6
Complete and single-copy	82.8	86.3	88.3	85.3	—
Complete and duplicated	13.4	9.8	8.3	10.4	—
**Chromosomes BUSCO completeness (%)**					
Complete	95.3	94.3	96.2	95.0	99.6
Complete and single-copy	92.3	91.5	93.8	91.8	—
Complete and duplicated	3.0	2.8	2.5	3.2	—
Size of repeat sequences (Mb)	514.2	534.1	489.3	503.6	2041.2
Total gene number	44156	49598	43845	44330	181929

Contigs identified as contaminants were removed based on blastn (v0.8.1) searches against a custom-built contaminant database, which includes *Solanum* plastid and mitochondrial sequences as well as bacterial sequences all downloaded from NCBI RefSeq release 218 (https://ftp.ncbi.nlm.nih.gov/genomes/refseq/).

Decontaminated scaffolds were anchored to chromosomes by mapping Hi-C reads to each haplotype set separately following the manufacturer’s recommended pipeline for Omni-C data (https://omni-c.readthedocs.io). Briefly, Hi-C reads were mapped using BWA-MEM (v0.7.17-r1188)^26^ then the mappings were parsed with *pairtools* (v0.3.0)^27^ followed by samtools (v1.3.1)^28^ to identify and extract valid pairs. Valid pairs were used to anchor and orient scaffolds into chromosomes using YaHS (v1.2a.1)^29^ and Juicebox Assembly Tools (v2.17.00)^30,31^.

Chromosomes 11 and 12 of haplotype 4 lacked ~20 Mbp and ~30 Mbp part of the pericentromeric region, respectively, and haplotype 1 contained two additional unplaced scaffolds (scaffold_22 and scaffold_23). Alignment of these scaffolds to reference genome (DM v6.1) and inspection of Hi-C contacts suggested that these scaffolds are the missing regions of chromosomes 11 and 12 in haplotype 4. Therefore, we remapped Hi-C reads and incorporated these two scaffolds in haplotype 4 using Juicebox Assembly Tools (v2.17.00).

The final scaffolded assembly size amounts to 3.3 Gbp, with individual haplotypes ranging between 762 and 888 Mb. As expected, one haplotype is highly similar to the DM haplotype, whereas other haplotypes can be more dissimilar (Fig. 1c). A comparison of Merqury k-mer spectra between the initial contigs and the scaffolded chromosomes (Fig. 1a) reveals that many apparent duplications in the contigs are resolved during scaffolding. A small proportion of sequences remains missing from the chromosomes and those can be found in the whole genome FASTA.

The haplotype assemblies were sequentially aligned using minimap2 (v2.28) and analyzed with SyRi (1.7.0) to identify syntenic regions and structural rearrangements which were visualized using plotsr (v1.1.1, Fig. 1d).

### Genome annotation

Repeat elements in the *S. tuberosum* cv. Désirée genome were identified using the Extensive *de novo* TE Annotator (EDTA, v2.2.1)^32^. Repetitive sequences cover 489–534 Mbp per haplotype, representing more than 70% of the genome (Table 2).

**Table 2:** Summary of genome annotations for each haplotype.

Type	haplotype 1	haplotype 2	haplotype 3	haplotype 4
**Repeat elements**				
DNA	46.6 Mbp (6.4%)	54.1 Mbp (7.4%)	44.6 Mbp (6.4%)	45.9 Mbp (6.5%)
Helitron	36.1 Mbp (5.0%)	38.0 Mbp (5.2%)	33.6 Mbp (4.8%)	42.3 Mbp (6.0%)
LINE	12.4 Mbp (1.7%)	8.1 Mbp (1.1%)	7.5 Mbp (1.1%)	8.1 Mbp (1.1%)
LTR	176.5 Mbp (24.4%)	188.0 Mbp (25.8%)	165.9 Mbp (23.7%)	193.6 Mbp (27.3%)
LTR/Copia	16.8 Mbp (2.3%)	19.3 Mbp (2.6%)	20.3 Mbp (2.9%)	23.9 Mbp (3.4%)
LTR/Gypsy	136.2 Mbp (18.9%)	133.6 Mbp (18.3%)	130.0 Mbp (18.6%)	102.8 Mbp (14.5%)
MITE	11.9 Mbp (1.6%)	10.2 Mbp (1.4%)	13.0 Mbp (1.9%)	10.6 Mbp (1.5%)
Other	72.8 Mbp (10.1%)	76.2 Mbp (10.4%)	69.7 Mbp (10.0%)	71.3 Mbp (10.1%)
SINE	5.1 Mbp (0.7%)	6.6 Mbp (0.9%)	4.7 Mbp (0.7%)	4.9 Mbp (0.7%)
Total	514.2 Mbp (71.2%)	534.1 Mbp (73.2%)	489.3 Mbp (70.1%)	503.6 Mbp (71.0%)
**Protein-coding genes**				
Total gene number	44156	49598	43845	44330
Total transcript number	67585	72597	66972	67185
Mean gene length (bp)	1695.85	1610.97	1687.71	1677.79
Mean CDS length (bp)	1062.59	1032.74	1060.23	1061.68
Mean exon number	5.28	5.04	5.31	5.28
Mean intron number	4.28	4.04	4.31	4.28
Complete BUSCO (%)	94.1%	93.3%	95.4%	93.7%
Single Omark HOGs	82.9%	82.5%	84.3%	82.8%
Duplicated Omark HOGs	11.6%	11.6%	11.5%	11.9%
Missing Omark HOGs	5.5%	5.9%	4.2%	5.4%
Mercator4 proteins annotated (%)	93.5%	93.5%	93.7%	93.5%
Mercator4 proteins classified (%)	50.5%	46.5%	50.7%	50.0%
Mercator4 bins occupied (%)	94.2%	93.9%	94.6%	94.3%

**Fig. 2:**
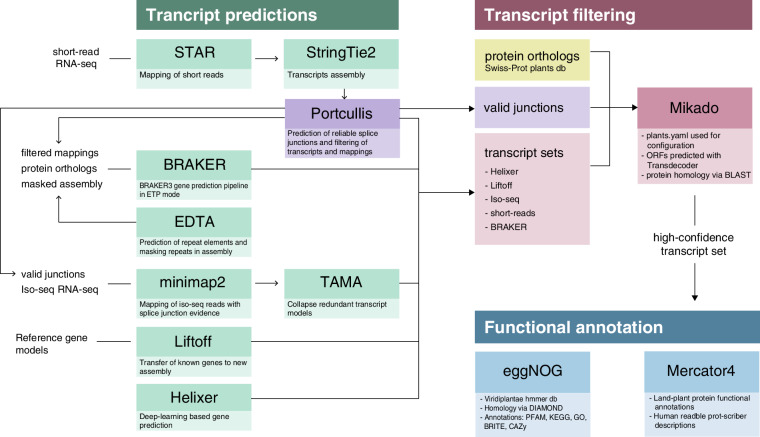
Workflow overview of *S. tuberosum* cv. Désirée genome annotation.

The prediction of protein-coding genes in the assembled *S. tuberosum* cv. Désirée was determined using five complementary approaches: *de novo*, homology-based, transcriptome-based, deep-learning, and reference-based predictions (Fig. 2). The annotation pipeline was run for each haplotype independently.

For transcriptome-based prediction, two methods were applied for short reads^33–40^ and Iso-Seq reads^41^, respectively. Short reads from multiple tissues were aligned to each haplotype using STAR (2.7.10a)^42^, and transcripts were assembled with StringTie2 (v2.2.1)^43^, followed by Portcullis (v1.2.4)^44^ for junction validation. Iso-Seq reads from five *S. tuberosum* cultivars were mapped to both haplotypes using minimap2 (v2.28)^45^, and transcripts were generated using IsoQuant (v3.3.1)^46^ and TAMA Collapse (tc_version_date_2023_03_28)^47^.

BRAKER3 (v3.0.8)^48^ was used in ETP mode to predict gene models by integrating *de novo*, homology-based, and transcriptome-based predictions. Repeat masking of the assembly was performed with RepeatMasker (v4.1.2), using EDTA annotations. Protein sequences from OrthoDB (green plant orthologs) were provided as evidence, and short-read STAR alignments with invalid junctions removed were included.

Helixer (v0.3.3)^49,50^ was used for deep-learning-based gene prediction via its web interface (https://www.plabipd.de/helixer_main.html). Gene models from the *S. tuberosum* reference genome (DM v6.1, UniTato annotation) were transferred to the Désirée assembly using Liftoff (v1.6.3)^51^. All five transcript or gene model sets were consolidated using Mikado (v2.3.4)^52^ and UniProt plants database (review version 2024_04_22)^53^ to generate a non-redundant set of transcripts. Protein-coding gene completeness was assessed using BUSCO (Tables 2, v5.4.7, solanales_odb10 dataset) and OMArk (v0.3.0, omamer v2.0.2)^54^. The quality of protein-coding gene annotations was assessed using PSAURON (v 1.0.4)^55^ and results were added to the GFF3 annotation file.

Transcriptomic data used for gene annotation was downloaded from public repositories: SRA under accessions SRP548344^34^, SRP545376^35^, SRP315827^41^, SRP358130^33^, SRP556848^36^ and SRP547875^37^; the Gene Expression Omnibus (GEO) under accession GSE232028^39^; and the National Genomics Data Center (NGDC) under accession CRA006012^38^. Existing gene models used in the gene annotation pipeline were downloaded from https://unitato.nib.si and https://spuddb.uga.edu.

The predicted protein-coding genes were functionally annotated using EggNOG Mapper (v2.1.11)^56^ with the EggNOG database (version 5.0.2)^57^ for the Viridiplantae subset. This included categories such as gene names, Gene Ontologies (GOs), enzyme functions (EC), and KEGG pathways, reactions, and modules, along with CAZy families, PFAM domains, and more. Additionally, functional land-plant protein annotations were predicted using Mercator4 (v7)^58^ via the web platform (https://www.plabipd.de/mercator_main.html). Annotations from EggNOG and Mercator4 were combined into the final GFF3 annotation file.

Orthologous groups between haplotypes and UniTato genes were identified using OrthoFinder (v2.5.5)^59^ using default setting. Across haplotypes, 55.3% of orthogroups contained genes from all four haplotypes, 22.9% from three haplotypes, 19.2% from two haplotypes, and 2.7% from a single haplotype. This is in line with the haplotype-resolved potato genome assemblies of cv. Atlantic^10^ and cv. Otava^11^. When comparing the Désirée annotation to UniTato, 17.24% of genes were specific to the Désirée annotation.

## Data Records

The raw sequencing data, including Illumina Hi-C, Illumina paired-end, PacBio HiFi, and ONT reads, have been deposited at the National Center for Biotechnology Information (NCBI) Sequence Read Archive (SRA) under BioProject number PRJNA1185028^18^. Plastid, mitochondrial and bacterial sequences used for removal of contaminant contigs were downloaded from NCBI RefSeq release 218. The genome assemblies of the four haplotypes have been submitted to NCBI GenBank under the umbrella BioProject accession PRJNA1217011^60–64^. The assembled genome, including annotations, methylation profile and identified orthologs, is hosted in a Zenodo repository under 10.5281/zenodo.15282553^65^ and is also accessible via an interactive genome browser at https://desiree.nib.si.

## Technical Validation

We assessed the assembly quality and completeness using DNA sequencing read mapping, CRAQ, BUSCO analysis, and Merqury k-mer based evaluation. Illumina reads were mapped with BWA (v0.7.17), while PacBio and ONT reads were aligned using minimap2 (v2.28). Mapping rates were 99.90%, 100.00%, and 99.74% for Illumina paired-end, PacBio, and ONT reads, respectively. CRAQ (v1.0.9)^66^ analysis of PacBio and Illumina mappings yielded a regional AQI of 96.3 and an overall AQI of 97.5, classifying the assembly as reference quality (AQI > 90). Assembly completeness was assessed with BUSCO (v5.4.7) using the solanales_odb10 lineage database, identifying 5930 (99.6%) of the 5950 BUSCO orthologous groups in both the whole genome and chromosome-only assemblies (Table 1). Merqury (v1.3) analysis, using a Meryl (v1.3) database constructed from Illumina reads, estimated genome completeness at 98.57% for the whole genome and 95.73% for the chromosomes. The estimated QV values were 54.30 and 58.53 for the whole genome and chromosomes, respectively.

**Fig. 3:**
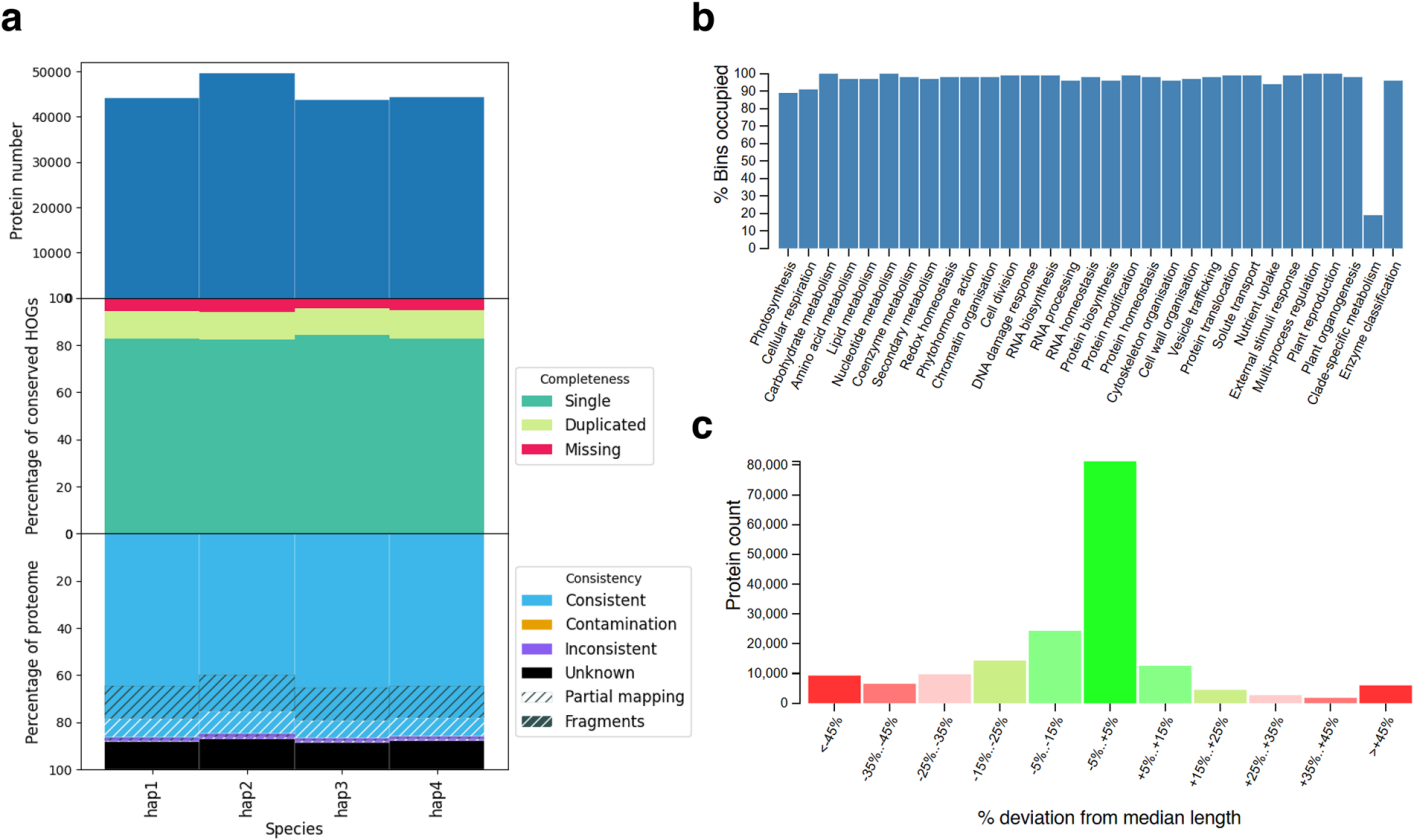
Validation of gene annotation. (**a**) OMArk quality assessment showing consistency, completeness and count of proteins across all four haplotypes. (**b**) Histogram showing the percentage of Mercator4 functional bins occupied by the Désirée proteins. (**c**) Histogram displaying the distribution of proteins grouped by their percentage deviation from the median protein length.

Completeness of gene annotation was assessed using OMArk (v0.3.0, omamer v2.0.2), BUSCO (v5.4.7) and Mercator4 (v7). OMArk analysis demonstrated that our annotation captured 94.1%–94.6% of Hierarchical Orthologous Groups (HOGs) per haplotype, with duplication rates ranging from 11.5% to 11.9% (Fig. 3a). When combining genes from all haplotypes, the proportion of complete HOGs reaches 99.3%, meaning that not all conserved genes are present in all haplotypes. Similarly, BUSCO analysis reported a haplotype completeness range of 93.3%–95.4% (Table 2), while the whole genome annotation achieved 99.4% completeness. Protein classification via Mercator4 revealed that 93.9%–94.6% of Mercator bins were occupied per haplotype, increasing to 97.5% when combining all proteins (Table 2). As expected, the Mercator bin with the largest proportion of missing proteins was associated with clade-specific metabolism (Fig. 3b). Additionally, the classified proteins showed no significant deviation from the median protein length, confirming consistency in annotation quality (Fig. 3c).

## Usage Notes

The presented Désirée genome assembly is of high contiguity, completeness and phasing quality and presents a valuable resource for haplotype-aware transcriptomics, proteomics and epigenomics analyses. The transfer of UniTato annotations^67^ provides translation of gene identifiers from the DM to the Désirée genome. The RNA-seq datasets used to supplement gene model annotation are predominantly from mature leaf and root tissue, thus genes specifically expressed in other tissue and developmental stages may not be fully captured in the current annotation.

The genome was produced from a plant propagated in tissue culture for over a decade. A recent pangenome study^3^ found that *in vitro* propagated plants of the *Solanum* section Petota have greater numbers of TEs in their genomes. While this seems to hold for LTR elements and DNA transposons in the Désirée genome, overall TE expansion is not evident. Examining the DNA methylation profile available in the Désirée genome browser might provide more insight into specific transposable element expansion in this cultivar.

Recently, efforts were made to generate potato pangenomes^3,10^. However, the number of included phased tetraploid genomes is still limited. Including Désirée and more phased tetraploid genomes will improve the completeness of potato pangenome. This will bridge knowledge gaps in potato genomics and give potato breeders a powerful toolkit for developing more resilient and productive cultivars.

## Data Availability

The code, scripts and command-line tool commands used for genome assembly, annotation and quality control are freely available in the GitHub repository https://github.com/NIB-SI/desiree-genome.

## References

[1] Yang, X. *et al*. The gap-free potato genome assembly reveals large tandem gene clusters of agronomical importance in highly repeated genomic regions. *Molecular Plant* **16**, 314–317 (2023). 36528795 10.1016/j.molp.2022.12.010

[2] Pham, G. M. *et al*. Construction of a chromosome-scale long-read reference genome assembly for potato. *GigaScience* **9**, giaa100 (2020). 32964225 10.1093/gigascience/giaa100 PMC7509475

[3] Bozan, I. *et al*. Pangenome analyses reveal impact of transposable elements and ploidy on the evolution of potato species. *Proceedings of the National Academy of Sciences* **120**, e2211117120 (2023). 10.1073/pnas.2211117120 PMC10401005 37487084

[4] van Berloo, R., Hutten, R. C. B., van Eck, H. J. & Visser, R. G. F. An Online Potato Pedigree Database Resource. *Potato Res.* **50**, 45–57 (2007).

[5] The European Cultivated Potato Database. https://www.europotato.org/varieties/view/Desiree-E.

[6] Tomaž, Š. *et al*. A mini-TGA protein modulates gene expression through heterogeneous association with transcription factors. *Plant Physiology* **191**, 1934–1952 (2023). 36517238 10.1093/plphys/kiac579 PMC10022624

[7] Halim, V. A. *et al*. PAMP-induced defense responses in potato require both salicylic acid and jasmonic acid. *The Plant Journal* **57**, 230–242 (2009). 18801014 10.1111/j.1365-313X.2008.03688.x

[8] Lukan, T. *et al*. CRISPR/Cas9-mediated fine-tuning of miRNA expression in tetraploid potato. *Horticulture Research* **9**, uhac147 (2022). 36072839 10.1093/hr/uhac147 PMC9437727

[9] Bao, Z. *et al*. Genome architecture and tetrasomic inheritance of autotetraploid potato. *Molecular Plant* **15**, 1211–1226 (2022). 35733345 10.1016/j.molp.2022.06.009

[10] Hoopes, G. *et al*. Phased, chromosome-scale genome assemblies of tetraploid potato reveal a complex genome, transcriptome, and predicted proteome landscape underpinning genetic diversity. *Molecular Plant* **15**, 520–536 (2022). 35026436 10.1016/j.molp.2022.01.003

[11] Sun, H. *et al*. Chromosome-scale and haplotype-resolved genome assembly of a tetraploid potato cultivar. *Nat Genet* **54**, 342–348 (2022). 35241824 10.1038/s41588-022-01015-0 PMC8920897

[12] Serra Mari, R. *et al*. Haplotype-resolved assembly of a tetraploid potato genome using long reads and low-depth offspring data. *Genome Biology* **25**, 26 (2024). 38243222 10.1186/s13059-023-03160-z PMC10797741

[13] Reyes-Herrera, P. H. *et al*. Chromosome-scale genome assembly and annotation of the tetraploid potato cultivar Diacol Capiro adapted to the Andean region. *G3 Genes|Genomes|Genetics* **14**, jkae139 (2024). 39058924 10.1093/g3journal/jkae139 PMC11537804

[14] Freire, R. *et al*. Chromosome-scale reference genome assembly of a diploid potato clone derived from an elite variety. *G3 Genes|Genomes|Genetics* **11**, jkab330 (2021). 34534288 10.1093/g3journal/jkab330 PMC8664475

[15] van Lieshout, N. *et al*. Solyntus, the New Highly Contiguous Reference Genome for Potato (Solanum tuberosum). *G3 Genes|Genomes|Genetics* **10**, 3489–3495 (2020). 32759330 10.1534/g3.120.401550 PMC7534448

[16] Zhou, Q. *et al*. Haplotype-resolved genome analyses of a heterozygous diploid potato. *Nat Genet* **52**, 1018–1023 (2020). 32989320 10.1038/s41588-020-0699-x PMC7527274

[17] Doyle, J. DNA extraction by using DTAB-CTAB procedures. *Phytochemical Bulletin* **19**, 11–17 (1987).

[18] *NCBI Sequence Read Archive* http://identifiers.org/ncbi/insdc.sra:SRP544620 (2025).

[19] Cheng, H., Concepcion, G. T., Feng, X., Zhang, H. & Li, H. Haplotype-resolved de novo assembly using phased assembly graphs with hifiasm. *Nat Methods* **18**, 170–175 (2021). 33526886 10.1038/s41592-020-01056-5 PMC7961889

[20] Cheng, H. *et al*. Haplotype-resolved assembly of diploid genomes without parental data. *Nat Biotechnol* **40**, 1332–1335 (2022). 35332338 10.1038/s41587-022-01261-x PMC9464699

[21] Cheng, H., Asri, M., Lucas, J., Koren, S. & Li, H. Scalable telomere-to-telomere assembly for diploid and polyploid genomes with double graph. *Nat Methods* **21**, 967–970 (2024). 38730258 10.1038/s41592-024-02269-8 PMC11214949

[22] Camacho, C. *et al*. BLAST+: architecture and applications. *BMC Bioinformatics* **10**, 421 (2009). 20003500 10.1186/1471-2105-10-421 PMC2803857

[23] Wick, R. R., Schultz, M. B., Zobel, J. & Holt, K. E. Bandage: interactive visualization of de novo genome assemblies. *Bioinformatics* **31**, 3350–3352 (2015). 26099265 10.1093/bioinformatics/btv383 PMC4595904

[24] Rhie, A., Walenz, B. P., Koren, S. & Phillippy, A. M. Merqury: reference-free quality, completeness, and phasing assessment for genome assemblies. *Genome Biology* **21**, 245 (2020). 32928274 10.1186/s13059-020-02134-9 PMC7488777

[25] Manni, M., Berkeley, M. R., Seppey, M., Simão, F. A. & Zdobnov, E. M. BUSCO Update: Novel and Streamlined Workflows along with Broader and Deeper Phylogenetic Coverage for Scoring of Eukaryotic, Prokaryotic, and Viral Genomes. *Molecular Biology and Evolution* **38**, 4647–4654 (2021). 34320186 10.1093/molbev/msab199 PMC8476166

[26] Li, H. Aligning sequence reads, clone sequences and assembly contigs with BWA-MEM. *arXiv:1303.3997 [q-bio]* (2013).

[27] Open2C. *et al*. Pairtools: From sequencing data to chromosome contacts. *PLOS Computational Biology* **20**, e1012164 (2024). 38809952 10.1371/journal.pcbi.1012164 PMC11164360

[28] Danecek, P. *et al*. Twelve years of SAMtools and BCFtools. *GigaScience* **10**, giab008 (2021). 33590861 10.1093/gigascience/giab008 PMC7931819

[29] Zhou, C., McCarthy, S. A. & Durbin, R. YaHS: yet another Hi-C scaffolding tool. *Bioinformatics* **39**, btac808 (2023). 36525368 10.1093/bioinformatics/btac808 PMC9848053

[30] Dudchenko, O. *et al*. The Juicebox Assembly Tools module facilitates de novo assembly of mammalian genomes with chromosome-length scaffolds for under $1000. 254797 Preprint at 10.1101/254797 (2018).

[31] Juicebox Provides a Visualization System for Hi-C Contact Maps with Unlimited Zoom. *Cell Systems* **3**, 99–101 (2016). 10.1016/j.cels.2015.07.012 PMC5596920 27467250

[32] Ou, S. *et al*. Benchmarking transposable element annotation methods for creation of a streamlined, comprehensive pipeline. *Genome Biology* **20**, 275 (2019). 31843001 10.1186/s13059-019-1905-y PMC6913007

[33] *NCBI Sequence Read Archive* http://identifiers.org/ncbi/insdc.sra:SRP358130 (2022).

[34] *NCBI Sequence Read Archive* http://identifiers.org/ncbi/insdc.sra:SRP548344 (2025).

[35] *NCBI Sequence Read Archive* http://identifiers.org/ncbi/insdc.sra:SRP545376 (2025).

[36] *NCBI Sequence Read Archive* http://identifiers.org/ncbi/insdc.sra:SRP556848 (2025).

[37] *NCBI Sequence Read Archive* http://identifiers.org/ncbi/insdc.sra:SRP547875 (2025).

[38] *NGDC Genome Sequence Archive* https://ngdc.cncb.ac.cn/gsa/browse/CRA006012 (2024).

[39] Petek, M., Godec, T., Stare, K., Lukan, T. & Gruden, K. *GEO* http://identifiers.org/geo:GSE232028 (2025).

[40] Lukan, T. *et al.* An ERF transcription factor StPTI5, a novel regulator of endophyte community maintenance in potato. Preprint at 10.1101/2025.04.24.650297 (2025). 10.1111/nph.71240 PMC13326511 42178776

[41] *NCBI Sequence Read Archive* http://identifiers.org/ncbi/insdc.sra:SRP315827 (2022).

[42] Dobin, A. *et al*. STAR: ultrafast universal RNA-seq aligner. *Bioinformatics* **29**, 15–21 (2013). 23104886 10.1093/bioinformatics/bts635 PMC3530905

[43] Shumate, A., Wong, B., Pertea, G. & Pertea, M. Improved transcriptome assembly using a hybrid of long and short reads with StringTie. *PLOS Computational Biology* **18**, e1009730 (2022). 35648784 10.1371/journal.pcbi.1009730 PMC9191730

[44] Mapleson, D., Venturini, L. & Swarbreck, D. EI-CoreBioinformatics/portcullis. EI-CoreBioinformatics (2024).

[45] Li, H. New strategies to improve minimap2 alignment accuracy. *Bioinformatics* **37**, 4572–4574 (2021). 34623391 10.1093/bioinformatics/btab705 PMC8652018

[46] Prjibelski, A. D. *et al*. Accurate isoform discovery with IsoQuant using long reads. *Nat Biotechnol* **41**, 915–918 (2023). 36593406 10.1038/s41587-022-01565-y PMC10344776

[47] Kuo, R. I. *et al*. Illuminating the dark side of the human transcriptome with long read transcript sequencing. *BMC Genomics* **21**, 751 (2020). 33126848 10.1186/s12864-020-07123-7 PMC7596999

[48] Gabriel, L. *et al*. BRAKER3: Fully automated genome annotation using RNA-seq and protein evidence with GeneMark-ETP, AUGUSTUS, and TSEBRA. *Genome Res.* **34**, 769–777 (2024). 38866550 10.1101/gr.278090.123 PMC11216308

[49] Holst, F. *et al*. Helixer–de novo Prediction of Primary Eukaryotic Gene Models Combining Deep Learning and a Hidden Markov Model. 2023.02.06.527280 Preprint at 10.1101/2023.02.06.527280 (2023). 10.1038/s41592-025-02939-1 PMC13076211 41286201

[50] Stiehler, F. *et al*. Helixer: cross-species gene annotation of large eukaryotic genomes using deep learning. *Bioinformatics* **36**, 5291–5298 (2021). 33325516 10.1093/bioinformatics/btaa1044 PMC8016489

[51] Shumate, A. & Salzberg, S. L. Liftoff: accurate mapping of gene annotations. *Bioinformatics* **37**, 1639–1643 (2021). 33320174 10.1093/bioinformatics/btaa1016 PMC8289374

[52] Venturini, L., Caim, S., Kaithakottil, G. G., Mapleson, D. L. & Swarbreck, D. Leveraging multiple transcriptome assembly methods for improved gene structure annotation. *GigaScience* **7**, giy093 (2018). 30052957 10.1093/gigascience/giy093 PMC6105091

[53] The UniProt Consortium. UniProt: the Universal Protein Knowledgebase in 2025. *Nucleic Acids Research* **53**, D609–D617 (2025). 39552041 10.1093/nar/gkae1010 PMC11701636

[54] Nevers, Y. *et al*. Quality assessment of gene repertoire annotations with OMArk. *Nat Biotechnol* 1–10 10.1038/s41587-024-02147-w (2024). 10.1038/s41587-024-02147-w PMC11738984 38383603

[55] Sommer, M. J., Zimin, A. V. & Salzberg, S. L. PSAURON: a tool for assessing protein annotation across a broad range of species. *NAR Genomics and Bioinformatics* **7**, lqae189 (2025). 39781514 10.1093/nargab/lqae189 PMC11704789

[56] Cantalapiedra, C. P., Hernández-Plaza, A., Letunic, I., Bork, P. & Huerta-Cepas, J. eggNOG-mapper v2: Functional Annotation, Orthology Assignments, and Domain Prediction at the Metagenomic Scale. *Molecular Biology and Evolution* **38**, 5825–5829 (2021). 34597405 10.1093/molbev/msab293 PMC8662613

[57] Huerta-Cepas, J. *et al*. eggNOG 5.0: a hierarchical, functionally and phylogenetically annotated orthology resource based on 5090 organisms and 2502 viruses. *Nucleic Acids Research* **47**, D309–D314 (2019). 30418610 10.1093/nar/gky1085 PMC6324079

[58] MapMan4: A Refined Protein Classification and Annotation Framework Applicable to Multi-Omics Data Analysis. *Molecular Plant* **12**, 879–892 (2019). 10.1016/j.molp.2019.01.003 30639314

[59] Emms, D. M. & Kelly, S. OrthoFinder: phylogenetic orthology inference for comparative genomics. *Genome Biology* **20**, 238 (2019). 31727128 10.1186/s13059-019-1832-y PMC6857279

[60] Godec, T., Beier, S., Usadel, B., Gruden, K. & Petek, M. Solanum tuberosum genome sequencing. Genbank https://identifiers.org/ncbi/bioproject:PRJNA1217011.

[61] De_hap1_v1 assembly for Solanum tuberosum. *Genbank.* https://identifiers.org/ncbi/insdc.gca:GCA_049996075.1 (2025).

[62] De_hap2_v1 assembly for Solanum tuberosum. *Genbank.* https://identifiers.org/ncbi/insdc.gca:GCA_049996055.1 (2025).

[63] De_hap3_v1 assembly for Solanum tuberosum. *Genbank.* https://identifiers.org/ncbi/insdc.gca:GCA_049996115.1 (2025).

[64] De_hap4_v1 assembly for Solanum tuberosum. *Genbank.* https://identifiers.org/ncbi/insdc.gca:GCA_049996095.1 (2025).

[65] Godec, T. & Petek, M. Haplotype-resolved genome assembly of the tetraploid potato cultivar Désirée. *Zenodo*10.5281/zenodo.15282553 (2025). 10.1038/s41597-025-05372-3 PMC12181383 40541992

[66] Li, K., Xu, P., Wang, J., Yi, X. & Jiao, Y. Identification of errors in draft genome assemblies at single-nucleotide resolution for quality assessment and improvement. *Nat Commun* **14**, 6556 (2023). 37848433 10.1038/s41467-023-42336-w PMC10582259

[67] Zagorščak, M. *et al*. Evidence-based unification of potato gene models with the UniTato collaborative genome browser. *Front. Plant Sci*. **15** (2024). 10.3389/fpls.2024.1352253 PMC11196761 38919818

